# Philip Schieffelin & Co’s Drugs

**Published:** 1849-09

**Authors:** 


*Philip Schieffelin & Co.'s Drugs.*—We would invite the attention of our readers to the card of Philip Schieffelin & Co., inserted in the advertising sheet of this No. of this Journal.

This plan of preparing medicines is designed to secure practitioners and apothecaries against spurious or adulterated articles, which are far more extensively sold and used than many practitioners are aware of, notwitstanding all that has been said and written, of late, on the subject. Practitioners in the country, who are obliged to dispense medicines, will find their advantage in obtaining those supplied in this way. The character of the house from which these drugs emanate, is presumed to be a reliable guaranty of their purity and excellence. We observe among the proceedsing of the Ohio Medical Convention, held at Columbus on the 5th of June 1849, the following resolution pertaining to this subject :

"*Resolved*, That the thanks of the medical profession are due to the house of Philip Schieffelin & Co., of New York, for their efforts to furnish the community with pure drugs; and we recommend their extra medicines to the confidence of dealers and practitioners."

